# Real-Time Monitoring and Detection of Single-Cell Level Cytokine Secretion Using LSPR Technology

**DOI:** 10.3390/mi11010107

**Published:** 2020-01-19

**Authors:** Chen Zhu, Xi Luo, Wilfred Villariza Espulgar, Shohei Koyama, Atsushi Kumanogoh, Masato Saito, Hyota Takamatsu, Eiichi Tamiya

**Affiliations:** 1 Department of Applied Physics, Graduate School of Engineering, Osaka University, 2-1 Yamadaoka, Suita, Osaka 565-0871, Japan; chen@ap.eng.osaka-u.ac.jp (C.Z.); wilfred@ap.eng.osaka-u.ac.jp (W.V.E.); saitomasato@ap.eng.osaka-u.ac.jp (M.S.); 2 AIST-Osaka University Advanced Photonics and Biosensing Open Innovation Laboratory, AIST, 2-1 Yamadaoka, Suita, Osaka 565-0871, Japan; 3 Immunology Frontier Research Center, Graduate School of Medicine, Osaka University, 2-2 Yamadaoka, Suita, Osaka 565-0871, Japan; koyama@imed3.med.osaka-u.ac.jp (S.K.); kumanogo@imed3.med.osaka-u.ac.jp (A.K.); thyota@imed3.med.osaka-u.ac.jp (H.T.)

**Keywords:** localized surface plasmon resonance (LSPR) technology, Interleukin 6 (IL-6) detection, single cell trapping, single cell level immunoassay

## Abstract

Cytokine secretion researches have been a main focus of studies among the scientists in the recent decades for its outstanding contribution to clinical diagnostics. Localized surface plasmon resonance (LSPR) technology is one of the conventional methods utilized to analyze these issues, as it could provide fast, label-free and real-time monitoring of biomolecule binding events. However, numerous LSPR-based biosensors in the past are usually utilized to monitor the average performance of cell groups rather than single cells. Meanwhile, the complicated sensor structures will lead to the fabrication and economic budget problems. Thus, in this paper, we report a simple synergistic integration of the cell trapping of microwell chip and gold-capped nanopillar-structured cyclo-olefin-polymer (COP) film for single cell level Interleukin 6 (IL-6) detection. Here, in-situ cytokine secreted from the trapped cell can be directly observed and analyzed through the peak red-shift in the transmittance spectrum. The fabricated device also shows the potential to conduct the real-time monitoring which would greatly help us identify the viability and biological variation of the tested single cell.

## 1. Introduction

Cytokines are a broad and loose category of small immunological protein biomarkers secreted by the immune cells. They play a critical role in adjusting the cell signaling, cell differentiation and biological response in the human immune system, and are proven to be involved in cell autocrine, paracrine and endocrine signaling as immune-modulating agents [1,2,3,4]. Thus, the researches about cytokines have been a main focus of studies among the scientists in the recent decades. Among all the cytokines, IL-6 stands out for its outstanding contribution to clinical diagnosis and cell immunoassay. It is an interleukin that acts as both a pro-inflammatory cytokine, an anti-inflammatory myokine and also an important mediator of fever and acute phase responses [5]. In addition, the IL-6 is responsible for stimulating acute phase protein synthesis, as well as the production of neutrophils in the bone marrow. It supports the growth of B cells and is antagonistic to regulatory T cells [6,7,8]. Thus, the detection of IL-6 becomes our first target in this research.

The enzyme-linked immunosorbent assay (ELISA) is one of the most widely used conventional methods for cytokine detection recently. This conventional method allows sufficient quantification of target proteins via only a simple parallel array-type operation [9,10]. However, there still exists some weak points within this method. For example, the ELISA requires secondary antibodies that bind with target analytes and complex sample labeling, which make it time consuming [11]. To deal with the weak points of ELISA, the scientists report an improved technology named enzyme linked immunospot (ELISPOT) assay. The ELISPOT is a type of method that focuses on the quantitatively high-throughput measuring of single cell level cytokine secretion with much higher sensitivity [12]. Although numerous advantages could be provided by ELISPOT, there still remains a huge concern that the personal counting errors during the experiments will have an impact on the final results [13,14,15]. Another conventional method, fluorescence-based single cell intensity detection, requires multiple times fluorescence dyes staining which is also time consuming and complex. At the same time, a large amount of sample volume is needed, which has a great impact on the saving of precious samples especially in clinical applications [16,17,18]. Aside from the technologies mentioned above, the localized surface plasmon resonance (LSPR) is another widely used method for fast, label-free and real-time monitoring of biomolecule binding events [19,20,21,22]. The LSPR is a plasmonic phenomenon that arises around nanoscale structures or nanoparticles of noble metals when light is illuminated onto a nanoscale-featured sensing surface [21,23,24,25]. It will occur when the natural frequency of the oscillating conduction electrons of the conductive metal nanoparticles matches the incident light frequency, causing resonant oscillations of electrons [23,26,27]. Currently, LSPR-based biosensors utilize the biomolecular interactions that lead to the change of the refractive index (RI) in the vicinity of the sensing surface to conduct the spontaneous detection, which is proved highly significant for diagnostic and point-of-care testing (POCT) purposes [21,28]. Especially for detecting the antibody–antigen interactions, any changes of RI could result in a sensitive response in the LSPR-induced light absorption spectrum, which is beneficial for quantitative analysis. Until now, numerous LSPR-based biosensors integrated with microfluidics have been proposed for therapeutic applications and potential to realize the portable detecting platforms [29,30,31].

In order to fabricate the cost-effective LSPR-based biosensors, a cheap mass production technology such as nanoimprinting is extremely needed. Thus, in the previous research, we have already reported the fabrication of a nanoimprinted gold-capped nanopillar structures on cyclo-olefin-polymer (COP) for LSPR-based detections [4]. In this work, a microwell array and a gold-capped nanopillar structure were integrated into a simple analysis platform. Further research showed that microwell arrays could provide the relatively high single cell occupancy capability and meanwhile supply a suitable environment for long-time and real-time monitoring. Thousands of individual cells could be trapped and monitored within trap sites through simple gravitational sedimentation. The regular cell migration and cell-to-cell interactions could also be avoided by the trapping structure and well-to-well pitches [32,33,34,35]. In our proposed device, the edge area of the microwell structure was utilized to detect the RI change owing to the cytokine secretion and antibody binding.

In this research, fresh cultured IL-6 over-expressed Jurkat cells were utilized to evaluate the sensitivity and capability of our fabricated device. The cultured cells were directly trapped and started to release IL-6 which would immediately bind with the antibody on the surface of nanopillar-structured LSPR detection film without stimulation [6,36,37]. The result proved that our fabricated device has the potential to trap single cells reaching over 60% occupancy efficiency with relatively low cell concentrations and volumes, which is extremely significant in clinical diagnosis. Furthermore, the device shows the capability to detect the single cell transmittance spectrum peak red-shift caused by single cell cytokine secretions and a maximum of 1.8 nm peak shift was observed by our device through real-time cell monitoring. 

## 2. Materials and Methods 

### 2.1. Fabrication of Porous Alumina (PA) Mold

In this research, the nanoporous anodic alumina oxide (AAO) was first prepared as the mold for nanopillar structure formation on cyclo-olefin-polymer (COP) films. The whole fabrication process has already been reported in our previous report in detail [4]. Briefly, the AAO mold was fabricated using a two-step anodizing treatments. The first anodizing step was conducted under a constant voltage of 80 V for 1 h to generate an aluminum oxide layer on the polished aluminum plate. Afterwards, the layer was removed by sinking inside a mixer containing phosphoric acid (1.16%, *w*/*v*) and chromic acid (5%, *w*/*v*) at 60 °C. The second anodizing step was conducted under a constant voltage of 80 V for 1 min. Finally, the phosphoric acid etching was carried out for 13 min at 40 °C. Then, the treated mold was carefully dried by pure N_2_ gas and stored for further use.

### 2.2. Fabrication and Immobilization of Gold-Capped Nanopillar Structured Polymer Film

**Figure 1:**
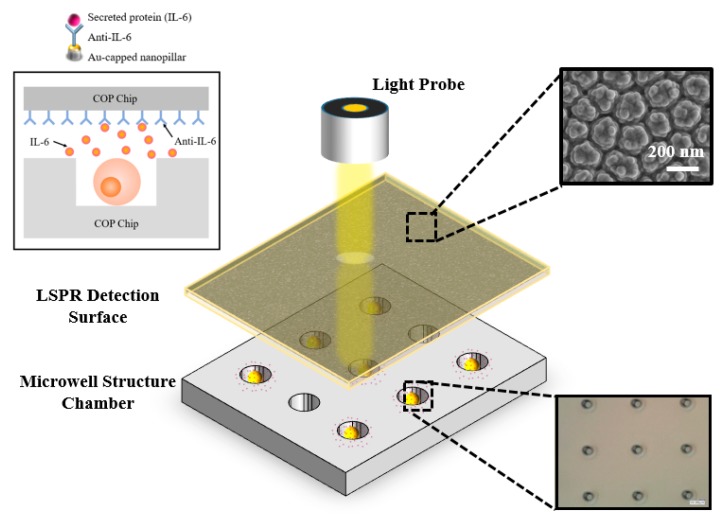
Schematic of the integrated localized surface plasmon resonance (LSPR) cytokine detection platform device.

The whole nanoimprinting procedure was conducted with X-300H (SCIVAX Corp., Kawasaki, Japan). A pressure of 0.83 MPa was applied for 1 min at 100 °C immediately after the PA-mold and COP (ZF-14-188) film were carefully arranged to the machine stage. Afterwards, the temperature was increased into 160 °C and the pressure was increased to 2 MPa for 10 min. Next, the pressure was released and the whole stage was cooling down to 80 °C. The processed COP film was carefully peeled off from the PA-mold. Next, the oxygen plasma etching was performed on the treated COP film to create an uneven structure on the surface of the nanopillar. According to the mechanism, it was considered that the COP resin surface was irradiated with oxygen (oxygen radicals) in a high energy state which would combined with carbon constituting the COP resin, vaporized and decomposed as CO_2_ [38]. After 60 s oxygen plasma treatment, the surface of the pillar roughened as shown in the scanning electron microscope (SEM) image in Figure 1. The diameter of the larger size pillar ranged from 150–200 nm and the distance between pillars was ranged from 20–50 nm. In addition, due to the oxygen plasma treatment, several smaller pillars ranging from 30–50 nm were formed on the surface of larger pillars. Then, the transformed COP chip was sputtered with gold using the Compact Sputter machine (ULVAC ACS4000, Yokohama, Japan) to form a 35 nm layer of gold on the COP film surface. Afterwards, it came to the immobilization steps. First, the gold-sputtered COP chip was submerged into the 10 mL 10-carboxy-1decanethiol reagent for 30 min to from a self-assembled monolayer (SAM) layer on the COP film surface and carefully washed with 99.5% Ethanol followed by a drying step via pure N_2_. Next, 100 μL of mixed reagent which consist of 0.1 M N-Hydroxysuccinimide (NHS) and 0.4 M 1-ethyl-3-(3-dimethylaminopropyl) carbodiimide hydrochloride (WSC) was uniformly dripped onto the surface of the COP film for 10 min and followed by a washing step via phosphate buffer saline (PBS) for activation. Furthermore, 100 μL of 50 ng/mL Anti-IL-6 was uniformly dripped onto the film surface for 30 min to combine with the previously made SAM layer. Finally, 100 μL of 1% BSA was coated onto the COP film surface for 30 min to block the whole structure followed by a washing step with PBST (PBS with 0.05% Tween-20) and PBS.

### 2.3. Fabrication of Cell Trapping Micro-Well Structured Chip

To fabricate the cell trapping chip, a clean silicon wafer mold was necessary. About 5 mL SU-8 3025 (Microchem, Newton, MA, USA) was first spin-coated on the surface of a clean 4-inch diameter silicon wafer according to the data sheet provided by the manufacturer at around 3000 rpm for 30 s to form a 20 μm uniform SU-8 layer. Afterwards, the cured SU-8 layer was exposed under the previously prepared glass mask and ultraviolet (UV) light for 6 s using a mask aligner (Mikasa, MA-10, Tokyo, Japan). The exposed mold was then treated under post exposure bake (PEB) protocol at 95 °C for 4 min followed by a developing process. Finally, the mold was rinsed, dried and stored in a clean container for further use. A clean 2 mm thick COP chip was carefully covered on the surface of the fabricated silicon mold and transferred into the nanoimprint machine. The nanoimprint step was performed under 1000 N pressure for 10 min under 40 °C. Afterwards, the COP chip was carefully removed from the mold and the cell trapping chip was successfully fabricated with the well diameter ranging from 13–15 μm. When the diameter of the microwell was set to 10 μm or lower, almost no single cells could be trapped by our device. Besides, if the diameter of the microwell was set to 16 μm or higher, there would be a high possibility to have cells stacking problems. Thus, here in our research, we choose to use the 13 μm as the standard diameter of the microwells.

### 2.4. Sensitivity Evaluation of the Gold-Capped COP Chip

The evaluation of the gold-capped COP film sensitivity is a quite significant index for analyzing the utility of the chip. In our research, the transmittance spectrum of the gold-capped COP surface was measured with the microscope (OLYMPUS IX70) with a spectrometer. Different refractive index environments were first evaluated, such as H_2_O (n = 1.33), 1 M glucose (n = 1.35), ethylene glycol (n = 1.43) and glycerol (n = 1.47). The absorption spectrum peak red-shift which resulted by the LSPR phenomenon was observed and plotted. The slope of the curve (peak shift/refractive index) was determined as the bulk sensitivity of the fabricated COP film. In addition, different concentrations of the IL-6 reagents were measured as the positive group, and the IgA reagent was measured as the negative group. Followed by this protocol, the limitation of detection (LOD) for IL-6 could be calculated.

### 2.5. Single Cell Occupancy Capability Evaluation of the Trapping Chip

The fresh cultured Jurkat cells were utilized to measure the occupancy capability of the fabricated micro-well structure COP chip. The cell trapping chip was first surrounded by the silicon rubber sheet for better injection of cell suspensions. Next, the chip was washed with MilliQ water and vacuumed to remove the trapped bubbles. Afterwards, 100 μL of cultured Jurkat cell suspension (concentration is 1 × 10^5^ cells/mL) was carefully pipetted onto the surface of the chip and waited for 15 min for gravity sedimentation. After cell sedimentation, a syringe was utilized to provide suitable fluid power, which would help to pipe out the extra media and cells from the device to avoid the cell stacking problems, as demonstrated in Figure S2. Finally, 10 μL of CD31 conjugated with FITC was utilized for fluorescence staining and the chip was stored under 4 °C for 30 min before monitoring. Counting the single cell position using this fluorescence-based technique could also greatly reduce the possibility to treat the stacked cells as the single cells. Optical observation was performed directly using an inverted microscope (Olympus IX-71, Tokyo, Japan) with ×10 magnification.

### 2.6. Real-time Monitoring of Single Cell IL-6 Secretion Situation

Fresh cultured IL-6 over-expressed Jurkat cells which could release a relatively greater number of IL-6 cytokines used for real-time cell monitoring. All the experimental tools were washed and autoclaved in advance to avoid any contamination. The cells were first cultured in the common media: 89% Roswell Park Memorial Institute (RPMI) 1640 media, 10% Fetal Bovine Serum (FBS) and 1% penicillin. Next, the cultured cells were carefully dripped onto the surface of the trapping chip and we waited 15 min for sedimentation. The COP detection film was cut into 1 × 1 cm^2^ pieces before the LSPR measurement. Afterwards, the cut film was covered on the top of the trapping chip, bound tightly and the integrated device was carefully placed under the microscope (OLYMPUS IX70, Tokyo, Japan) for real-time observation. The cell spectrum data was recorded every 6 min until 54 min, and the peak shifts in the absorption peak wavelength were recorded and visualized in a bar graph.

## 3. Results and Discussion

### 3.1. Morphology Characterization of the COP Detection and Trapping Device

The whole procedure to fabricate the single cell cytokine secretion detection device is shown in Figure 1. The device is simply the combination between the nanopillar-structured COP detection film and thick COP cell trapping chip, which is easily fabricated and portable. Figure 1 also shows the scanning electron microscope (SEM) image of the pillar structure. The anodization conditions have already been optimized for ease of removing the nanoimprinted COP film from the mold and formation of the nanostructures. In this research, several different well diameters and depths were evaluated to find the optimized conditions. As the diameter of target Jurkat cells were ranging from 8–12 μm, the single cell occupancy efficiency became relatively low when the trapping well diameter was less than 10 μm or over 16 μm which would lead to a high possibility to trap none or multiple cells. Meanwhile, the well depth also has a high impact on the cell occupancy efficiency. The experiment showed that the well depth ranging from 15–20 μm was more suitable for cell trapping. Thus, in our research, we finally chose to use the 13 μm diameter and 19 μm depth as the standard data to fabricate the trapping chip. Figure 1 (bottom) demonstrates the whole cell trapping chip and micro-well structures. In the whole design, 5000 single cell trapping wells are fabricated within the 1 × 1 cm^2^ area for high-throughput research and the well pitch is adjusted to 100 μm to avoid any cell-to-cell contaminations.

### 3.2. Sensitivity Evaluation of the COP Film via Transmittance Spectrum Peak Red-Shift

**Figure 2:**
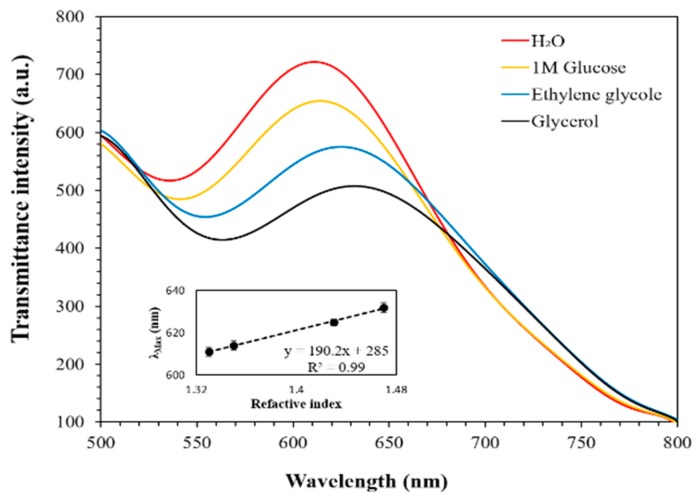
Transmittance LSPR peak wavelength shows red-shift response as observed in H_2_O (n = 1.33), 1 M glucose (n = 1.35), ethylene glycol (n = 1.43) and glycerol (n = 1.47) and individual wavelength shifts over the refractive index reveal the average sensitivity of the fabricated plasmonic device.

The LSPR transmittance peak shifts data of the fabricated COP detection films were recorded in several different refractive index reagents. Different reagents were uniformly dripped onto the surface of the COP films separately, and the peak shift data was illustrated in Figure 2.

According to the spectrum data, with the RI increases, the transmittance spectrum has a larger peak red-shift, which indicates that the fabricated COP detection film exhibits working plasmonic properties. Meanwhile, the bulk sensitivity of the COP detection film, the transmittance spectrum peak shifts are plotted in Figure 2, which shows a slope of 190.2 nm/RIU (Refractive index unit). The sensitivity results clearly claim that the fabricated COP detection film has higher sensitivity compared to the previously reported LSPR film [4], and could respond to the changes in RI with corresponding transmittance peak red-shifts, which is highly significant in LSPR-based single cell detection.

### 3.3. IL-6 Calibration Curve Detection Based on COP Detection Film

As IL-6 is our target cytokine in this research, it is necessary to evaluate the calibration curve for further detection. 100 μL of 10 ng/mL, 25 ng/mL, 50 ng/mL, 100 ng/mL and 200 ng/mL concentrations of IL-6 reagents are separately dripped onto the surface of the immobilized COP detection film for 30 min followed by a washing step. Afterwards, the COP detection films are observed under the microscope and the transmittance spectrum peak shifts are recorded and plotted.

**Figure 3:**
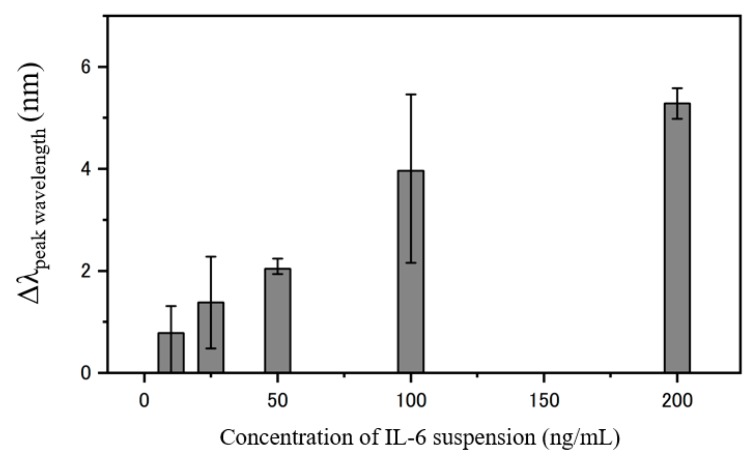
Calibration curve for concentrations 10 ng/mL, 25 ng/mL, 50 ng/mL, 100 ng/mL and 200 ng/mL IL-6 peak transmittance using fabricated plasmonic device.

Figure 3 shows the calibration curve obtained from the responses recorded by each different target concentrations. The result shows that the fabricated COP detection film has a linear response with a detection limitation of 10 ng/mL. 

### 3.4. Single Cell Occupancy Performance and Real-Time Monitoring of Single Cell Cytokine Secretion

**Figure 4:**
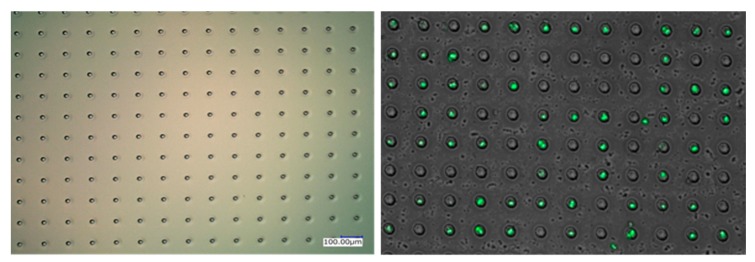
Evaluation of single cell occupancy efficiency using the fluorescence-based detection.

The single cell occupancy efficiency of the COP trapping chip was evaluated using the Jurkat cells (φ = 8–12 μm). In the experiment, 100 μL of the cell suspension (concentration is 1 × 10^5^ cells/mL) was dripped onto the surface of the trapping chip and we waited for 15 min for cell gravity sedimentation. Figure 4 shows the bright field image and also the fluorescence image of the trapped cells to clearly demonstrate that the cells are successfully isolated and trapped inside the micro-well structures. According to the observation result, our fabricated micro-well trapping devices could reach almost 60% single cell occupancy efficiency, as shown in Table 1. 

**Figure 5:**
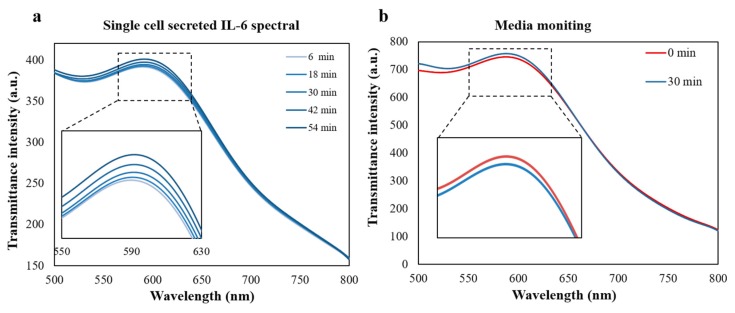
(**a**) Real-time transmittance spectrum observation of fresh-cultured IL-6 overexpressed single Jurkat cell using the fabricated plasmonic device during 54 min. (**b**) Negative control group to evaluate the effect of cell culture media to the LSPR transmittance spectrum detection after 30 min culturing.

Real-time monitoring of healthy single IL-6 over-expressed Jurkat cells were conducted for 54 min until the cells were dead. LSPR spectrum peak shift measurement of the single cell was recorded every 6 min and the data was illustrated in the Figure 5a. However, due to the limitation of our imaging system, it would take 30–40 s for single cell imaging scan and ~10 single cells were scanned at one time. This result to a scan limit of every 6 min and in the future, we will improve the techniques to conduct the single cell scan in a shorter time to realize the high-throughput analysis. In general, the transmittance peak tended to red-shift with time passing and remained the same until the final scan as the cells were already dead. The maximum transmittance spectrum peak red shift was 1.8 nm. At the same time, the control group experiment was conducted only using the new cell culture media. The result in Figure 5b shows that with the time passing, the cell culture media do not have great impact on the transmittance spectrum, which proves that the peak shift changes are resulted by the binding of IL-6 secreted by the single cell and immobilized anti-IL-6. Besides, we replaced the anti-IL-6 with anti-IgA during immobilization step as another negative control group, and found that there was also no obvious peak shift in the peak wavelength before and after binding, as demonstrated in Figure S1, which proved that the peak wavelength shift was caused by the specific binding of secreted IL-6 and anti-IL-6. This result indicates that our LSPR detection device has the potential to analyze the single cell level cytokine secretion situations.

## 4. Conclusions

In this research, a simple and portable LSPR detection device for single cell level cytokine secretion research is fabricated. With this device, over 3000 single cells could be isolated and trapped within the trapping sites at one time which is highly significant for the saving of precious samples especially in clinical medical diagnosis. In summary, our fabricated LSPR detection device could reach almost 60% single cell occupancy efficiency with relatively low sample concentrations and volumes. The bulk sensitivity of the device is found to be 190.2 nm/RIU which is proved capable for LSPR detection. A detection limitation of 10 ng/mL for anti-IL 6 is established using the fabricated film. Furthermore, real-time monitoring of healthy IL-6 over-expressed Jurkat cells are conducted on the device which shows a maximum of 1.8 nm peak red-shift during the one-hour detection period. The limitation of this study is not easy to realize the change of cell culture media for longer time monitoring. In the future, we will focus upon the improvement of the single cell trapping device which could help realize the longer time monitoring and also conduct the real patient sample experiments using the peripheral blood mononuclear cell (PBMC).

## Figures and Tables

**Table 1:** Evaluation of different occupancy efficiency.

Cell Occupancy Type	Single Cell	Double Cells	Multiple Cells
Occupancy Efficiency	~60%	~10%	~5%

## Supplementary Materials

The following are available online at https://www.mdpi.com/2072-666X/11/1/107/s1, Figure S1. Negative control group of anti-IgA to confirm the peak wavelength shift before and after binding. Figure S2. Cell trapping and washing procedures. After cell sedimentation, we use the syringe to provide fluid power which could help to pipe out the extra media and cells from the device to increase the single cell occupancy efficiency. Figure S3. Single cell peak wavelength shift corresponding to the time. Figure S4. Calibration curve for concentrations 1 ng/mL, 10 ng/mL, 25 ng/mL, 100 ng/mL, 200 ng/mL, 500 ng/mL, 750 ng/mL and 1000 ng/mL IL-6 peak wavelength using fabricated plasmonic device in dry conditions (dry with pure N_2_ gas).
