# Supplementary material for: Real-Time Monitoring and Detection of Single-Cell Level Cytokine Secretion Using LSPR Technology
Source: Micromachines (Basel). 2020 Jan 19;11(1):107. doi: 10.3390/mi11010107 (PMC7019717; doi:10.3390/mi11010107)
Supplement: Supplementary file 1 [file micromachines-11-00107-s001.pdf]

Supplementary Materials: Real-time Monitoring and Detection of Single-cell Level Cytokine Secretion using LSPR Technology

Chen Zhu, Xi Luo, Wilfred Villariza Espulgar, Shohei Koyama, Atsushi Kumanogoh, Masato Saito, Hyota Takamatsu and Eiichi Tamiya

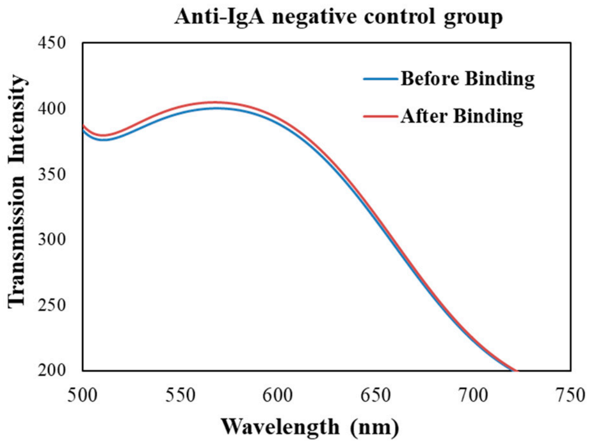

Figure S1. Negative control group of anti-IgA to confirm the peak wavelength shift before and after binding.

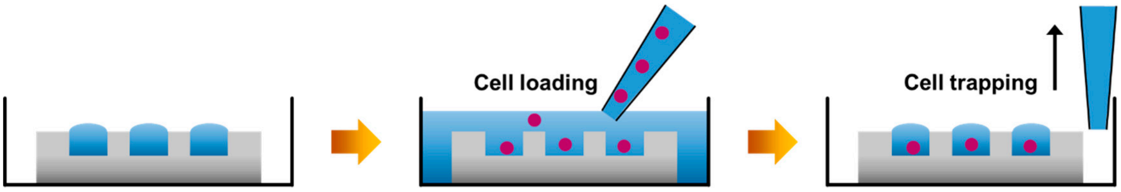

Figure S2. Cell trapping and washing procedures. After cell sedimentation, we use the syringe to provide fluid power which could help to pipe out the extra media and cells from the device to increase the single cell occupancy efficiency.

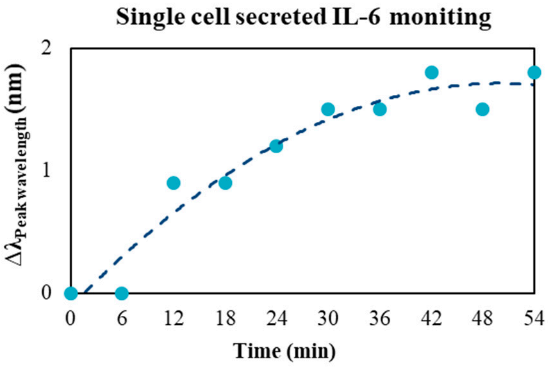

Figure S3. Single cell peak wavelength shift corresponding to the time.

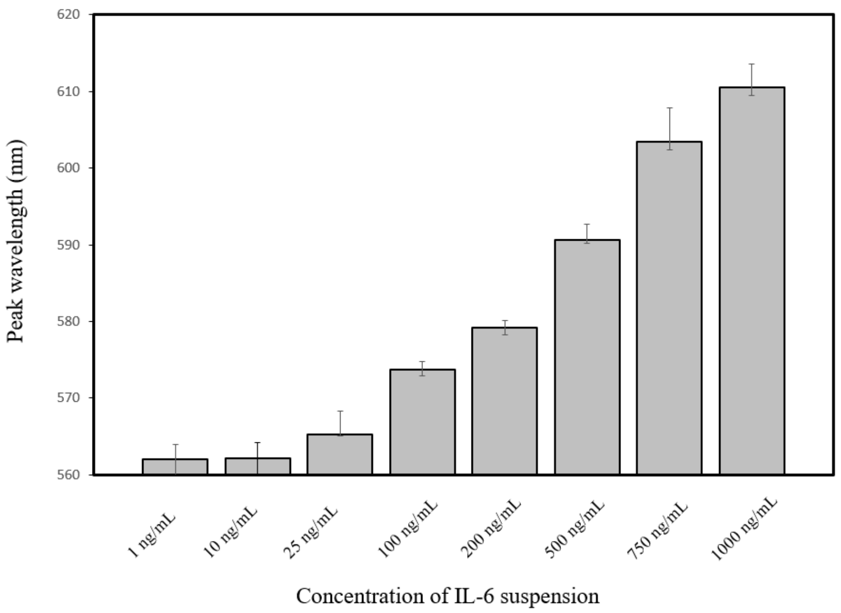

Figure S4. Calibration curve for concentrations 1 ng/mL, 10 ng/mL, 25 ng/mL, 100 ng/mL, 200 ng/mL, 500 ng/mL, 750 ng/mL and 1000 ng/mL IL-6 peak wavelength using fabricated plasmonic device in dry conditions (dry with pure N_2 gas).
